# Could I-FABP Be an Early Marker of Celiac Disease in Children with Type 1 Diabetes? Retrospective Study from the Tertiary Reference Centre

**DOI:** 10.3390/nu14030414

**Published:** 2022-01-18

**Authors:** Agnieszka Ochocińska, Marta Wysocka-Mincewicz, Artur Groszek, Anna Rybak, Ewa Konopka, Joanna Beata Bierła, Ilona Trojanowska, Mieczysław Szalecki, Bożena Cukrowska

**Affiliations:** 1 Department of Biochemistry, Radioimmunology and Experimental Medicine, The Children’s Memorial Health Institute, Aleja Dzieci Polskich 20, 04-730 Warsaw, Poland; 2 Department of Endocrinology and Diabetology, The Children’s Memorial Health Institute, Aleja Dzieci Polskich 20, 04-730 Warsaw, Poland; m.wysocka@ipczd.pl (M.W.-M.); a.groszek@ipczd.pl (A.G.); m.szalecki@ipczd.pl (M.S.); 3 Department of Gastroenterology, Great Ormond Street Hospital NHS Trust, Great Ormond Street, London WC1N 3JH, UK; anna.rybak@gosh.nhs.uk; 4 Department of Pathomorphology, The Children’s Memorial Health Institute, Aleja Dzieci Polskich 20, 04-730 Warsaw, Poland; e.konopka@ipczd.pl (E.K.); j.bierla@ipczd.pl (J.B.B.); i.trojanowska@ipczd.pl (I.T.); b.cukrowska@ipczd.pl (B.C.); 5 Collegium Medicum, Jan Kochanowski University, Aleja IX Wieków Kielce 19A, 25-317 Kielce, Poland

**Keywords:** type 1 diabetes, celiac disease, biomarker, intestinal fatty acid binding protein, impaired epithelial barrier, I-FABP, gluten-free diet, intestinal barrier

## Abstract

Patients with type 1 diabetes (T1D) are at higher risk of celiac disease (CD). Recently, intestinal fatty acid binding protein (I-FABP) has been shown to be a serological biomarker of impaired intestinal barrier in CD. Thus, the aim of this study was to verify whether I-FABP could be an early marker of CD in pediatric T1D patients. I-FABP was measured in sera of patients with T1D (*n* = 156), active CD (*n* = 38), T1D with active CD (T1D-CD, *n*= 51), and age-matched healthy children (*n* = 55). Additionally, I-FABP was determined in T1D patients with negative CD serology at least one year before CD diagnosis (T1D-CD-1, *n* = 22), in CD patients on a gluten-free diet (CD-GFD, *n* = 36), and T1D-CD patients on GFD (T1D-CD-GFD, *n* = 39). Sera were tested using immunoenzymatic assay. Significantly increased levels of I-FABP were found in the T1D, active CD, and T1D-CD groups (1153 ± 665, 1104 ± 916, and 1208 ± 878, respectively) in comparison to healthy with controls (485 ± 416, *p* < 0.05). GFD induced a significant decrease in I-FABP levels in CD and T1D-CD groups (510 ± 492 and 548 ± 439, respectively). Interestingly, in T1D-CD-1 and T1D, I-FABP levels were comparable (833 ± 369 vs. 1153 ± 665), and significantly increased in relation to healthy controls and T1D-CD values on GFD. The results indicate that the epithelial barrier is disrupted in T1D patients independently of CD development; therefore, I-FABP cannot serve as an early marker of CD in T1D patients. Although GFD can improve epithelial recovery, the question remains as to whether GFD could exert beneficial effects on the intestinal barrier in early stages of T1D.

## 1. Introduction

Type 1 diabetes (T1D) is a multifactorial and complex autoimmune disease. Its comorbidity with other autoimmune diseases, including celiac disease (CD), is well established. The co-diagnosis of CD affects from 2 to 16% of diabetic patients worldwide [1]. According to the latest Polish reports, the frequency of CD among T1D patients is now much higher than ten years ago (8.3% vs. 5.7%) and higher in girls (13.9%) than boys (4.9%), which is in line with previous reports by Cerutti et al. about the higher risk of having both diseases for girls than for boys [2,3].

The relationship between T1D and CD is being intensively studied [4]. The human leukocyte antigen (HLA) analysis showed that haplotypes occurring in almost all patients with CD (HLA-DQ2 and HLA-DQ8) exist in the majority of patients with T1D, which confirms the hypothesis concerning the common genetic pathogenesis of both diseases [5,6]. Common genetic features of T1D and CD were also documented in the genome-wide association study (GWAS), suggesting the role of impaired mucosal barrier function in the etiopathogenesis of both diseases [7]. In the altered gut barrier, non-competent intercellular junctions allow antigens, derived from the food or intestinal microbiota, to enter the circulation and activate the immune system into upregulated autoimmune responses [8].

Although the coexistence of CD and T1D is well known, the diagnosis of CD among diabetic patients remains a clinical challenge due to the fact that 60% to 70% of T1D children present with asymptomatic or non-classical CD [2]. Prompt diagnosis is necessary because untreated CD disrupts the absorption of nutrients by damaging the small intestine, therefore resulting in an increased risk of hypoglycemia. Undiagnosed CD in T1D patients may result in poor glycemic control, leading to more complications and, in consequence, insufficient treatment of retinopathy, nephropathy or dyslipidemia. This is why all experts agree that screening tests for CD should be performed for all T1D patients [9]. Due to the high frequency of CD-specific HLA haplotypes in T1D, genetic HLA-DQ-2 and HLA-DQ8 testing, as a first-line screening, suggested by the European Society for Pediatric Gastroenterology Hepatology and Nutrition (ESPGHAN) [10], is questionable. For this reason, CD serological markers are recommended by the International Society for Pediatric and Adolescent Diabetes (ISPAD) for all T1D patients, independently of symptomatology [11].

Nevertheless, new biomarkers are still being sought to enable the detection of intestinal epithelial damage at earlier stages. Recently, intestinal fatty acid binding proteins (I-FABP) were presented as novel serological biomarkers of active CD [12,13,14]. Elevated concentrations of I-FABP were observed in patients with untreated CD. Moreover, treatment with a gluten-free diet (GFD) induced the rapid normalization of I-FABP levels [14,15,16].

I-FABP is described as a low-molecular-weight (14–15 kDa) water-soluble extracellular protein, a type of fatty acid binding protein [17]. This protein is expressed in the epithelial cells of the mucous layer of the small intestine, and, in cases of epithelium damage, its increased concentration is observed in the blood [18,19].

Thus, the aim of our study was to assess whether I-FABP could be an early marker of CD in children with T1D.

## 2. Materials and Methods

### 2.1. Patients and Study Design

We performed an analysis of a retrospective research group collected prospectively as part of screening and subjected to post hoc analysis after the onset of celiac disease.

The study involved children with T1D and/or CD hospitalized in the Children’s Memorial Health Institute in Warsaw (Poland) in the period between 2012 and 2018. A case-control study was performed on patients with T1D (*n* = 156), with T1D and active CD (T1D-CD, *n* = 51), and with active CD only (*n* = 38), who were randomly chosen from this database. The patients’ characteristics are presented in Table 1. T1D patients who displayed no CD serological markers at diagnosis were annually serologically screened for CD, and a subgroup of patients who were eventually diagnosed with CD but had only T1D one year prior to diagnosis (T1D-CD-1 group, *n* = 22) were selected. In addition, among patients with T1D-CD and CD, a subgroup of patients who had been following GFD for at least 6 months was distinguished (CD-GFD, *n* = 36; T1D-CD-GFD, *n* = 39). Healthy children formed the control group (HC, *n* = 55).

T1D was diagnosed according to the recommendations of the ISPAD [11,20], and CD according to the ESPGHAN criteria [10]. Routine serological tests necessary to perform a diagnosis were performed in Department of Biochemistry, Radioimmunology and Experimental Medicine, and Department of Pathomorphology at The Children’s Memorial Health Institute (for T1D: anti-glutamic decarboxylase (anti-GAD), anti-tyrosine phosphatase (anti-IA2), anti-islet cell (ICA) antibodies, and for CD: serum anti-tissue transglutaminase antibody (anti-tTg Ab)). Patients and control group with current inflammation, hypoxia, and coexisting diseases of other causes were excluded from the study. All healthy controls had negative serological screening test.

### 2.2. I-FABP Measurement

I-FABP was measured in the sera of patients and control group using an enzyme-linked immunosorbent assay (ELISA) kit (Hycult Biotech Inc., Wayne, PA, USA) according to the manufacturer’s instructions. Absorbance values were measured using a BioTek PowerWave Microplate Spectrophotometer at a wavelength of 450 nm. Results were expressed as mean ± standard deviation (SD) in picograms/milliliters (pg/mL).

### 2.3. Statistical Analysis

Data were analyzed using Statistica v.10.0 software (StatSoft, Inc., Tulsa, OK, USA). Standard deviations of means were used as descriptive statistics. Normal distribution was checked using the Shapiro–Wilk test and revealed non-normal distribution of data. Differences between two groups were tested by U Mann–Whitney test, and between three or more subgroups by Kruskal–Wallis ANOVA by Ranks for independent groups. If differences were significant, post hoc analysis using Dunn–Bonferroni test was then performed. Receiver operating characteristic (ROC) curves were used to obtain the specificity and sensitivity of serum I-FABP to distinguish diabetic patients with CD from those without CD.

Analysis of parameters in two time points were performed using Wilcoxon signed-rank test for dependent samples. In all tests, *p* values < 0.05 were considered significant.

### 2.4. Ethical Approval

The study was approved by the Local Ethics Committee from the Children’s Memorial Health Institute with written informed consent obtained from participants over 16 years of age and/or their legal representative, as appropriate.

## 3. Results

### 3.1. I-FABP Levels in Sera of T1D Patients without CD, Patients with Active CD, and Patients with T1DM and CD

**Figure 1:**
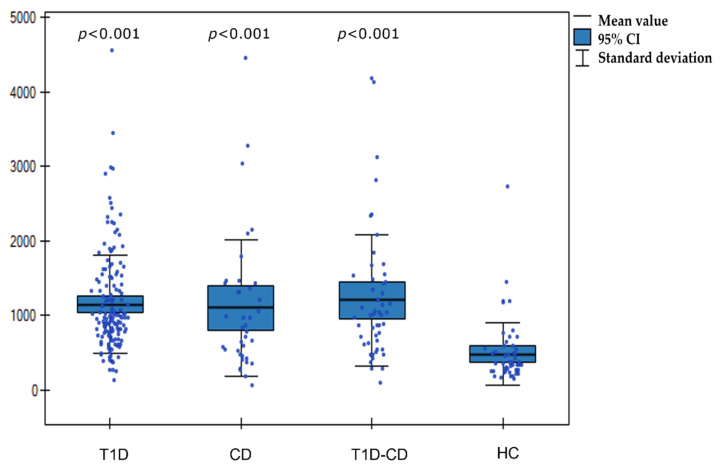
Boxplot chart illustrates the distribution of I-FABP concentrations in T1D, CD, T1D-CD, and HC. T1D—type 1 diabetes, CD —celiac disease, T1D-CD—type 1 diabetes and celiac disease, HC- healthy controls; *p* values were calculated by Kruskal–Wallis test.

There was a significant difference in I-FABP levels between the three study groups (T1D, active CD, and T1D-CD) and healthy controls: 1153 ± 665, 1104 ± 916, 1208 ± 878 vs. 485 ± 416 pg/mL, respectively; (*p* < 0.001) (Figure 1).

**Figure 2:**
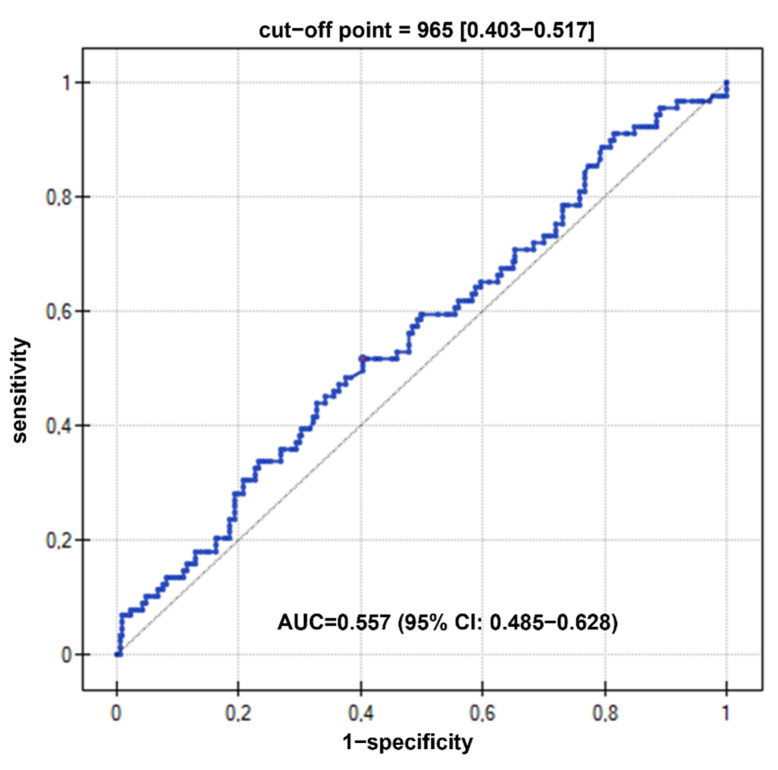
Diagnostic value of I-FABP as a marker of CD in T1D patients. AUC—area under curve, CI—confidence interval.

However, the statistical analysis did not show statistically significant differences between the groups (T1D, active CD, and T1D-CD patients). The ROC curve to detect CD in T1D patients revealed an area under curve (AUC) of 0.557 (95% confidence interval, CI: 0.485–0.628, *p* > 0.05) for I-FABP. A serum I-FABP concentration of >965 pg/mL was associated with the coexistence of both diseases, with a sensitivity of 51.7% and specificity of 59.7% (Figure 2).

### 3.2. The Effect of GFD on I-FABP Concentrations

**Figure 3:**
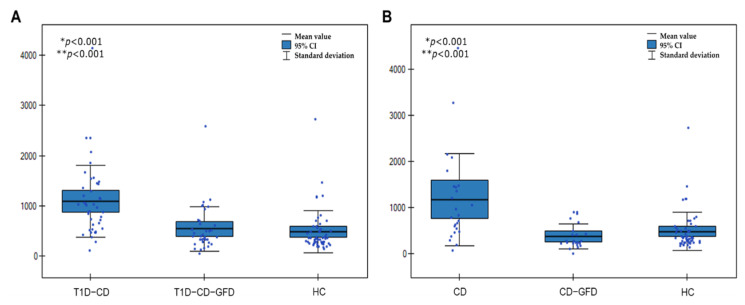
Boxplot chart illustrates the distribution of I-FABP concentrations in T1D-CD, T1D-CD-GFD, and HC patients (**A**) and CD, CD-GFD, and HC patients (**B**); *p* values were calculated by Wilcoxon signed-rank test; * *p*—T1D-CD (**A**) or CD (**B**) vs. HC, ** *p*—T1D-CD (**A**) or CD (**B**) vs. T1D-CD-GFD (**A**) or CD-GFD (**B**). T1D-CD—type 1 diabetes and celiac disease, T1D-CD-GFD—type 1 diabetes and celiac disease on gluten free diet, HC—healthy controls, CD—celiac disease, CD—celiac disease on gluten-free diet.

The serum level of I-FABP were substantially diminished in patients on GFD (Figure 3) and in both study groups (CD and T1D-CD), it reached values similar to those of the healthy control group. At least 6 months of GFD in T1D-CD patients induced a decrease in I-FABP concentration by 54.6% (from 1208 ± 878 to 548 ± 439 pg/mL). There were significant differences between patients with T1D-CD without dietetic treatment and on GFD (1208 ± 878 and 548 ± 439, respectively) as well as between the T1D-CD group and controls (1208 ± 878 pg/mL and 485 ± 416 pg/mL, respectively) (Figure 3A). In the case of CD patients, GFD resulted in a decrease in I-FABP concentration by 53.8% (from 1104 ± 916 to 510 ± 492 pg/mL). The differences between patients with active CD, before and after GFD treatment, as well as the control group, were statistically significant (1104 ± 916 vs. 510 ± 492 and 485 ± 416 pg/mL, respectively) (Figure 3B).

### 3.3. I-FABP Concentrations Prior to CD Diagnosis in Patients with Type 1 Diabetes

**Figure 4:**
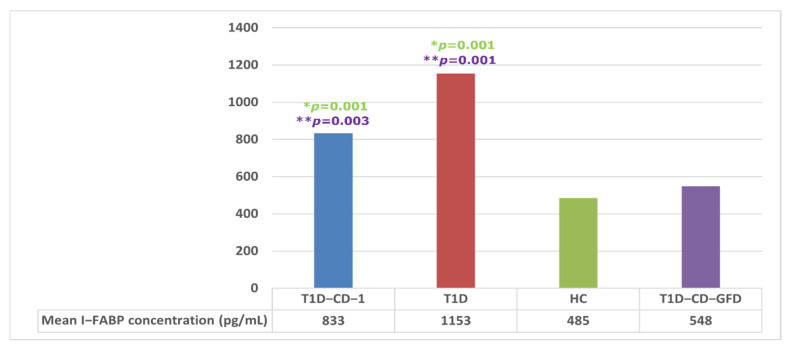
I-FABP concentrations in T1D-CD-1 group in relation to T1D, HC, and T1D-CD-GFD patients; *p* values were calculated by Wilcoxon signed-rank test; colors of *p* value fonts correspond to bars of study groups; * *p*—T1D-CD-1 or T1D vs. HC, ** *p*—T1D-CD-1 or T1D vs. T1D-CD-GFD. T1D-CD—type 1 diabetes and celiac disease patients with negative CD serology one year before CD diagnosis, T1D—type 1 diabetes, HC —healthy controls, T1D-CD-GFD—type 1 diabetes and celiac disease on gluten-free diet, I-FABP—intestinal fatty acid binding protein.

Mean value of I-FABP in the subgroup of patients with negative CD serology one year before CD diagnosis (T1D-CD-1) was not statistically significant different from that of T1D patients (833 ± 369 vs. 1153 ± 665 pg/mL, respectively). However, it differed significantly from the control group and T1D-CD patients after GFD treatment (833 ± 369 vs. 485 ± 416 and 548 ± 439 pg/mL, respectively) (Figure 4).

## 4. Discussion

The early detection of CD may be of crucial importance for patients with T1D, as numerous studies indicate that the treatment with GFD of patients with T1D and CD improves blood glucose control [21].

International experts on CD and T1D (ESPGHAN, ISPAD) agree on the necessity of screening diabetic patients with T1D for CD, but there is no consensus on the length of follow-up, testing frequency, and type of testing [10,11]. Due to the fact that HLA genotyping in patients with T1D is not sufficient to identify patients with an increased risk of CD, and that serological screening for CD allows the detection of the disease process only at an advanced stage, when severe histological changes are present in the small intestine, as well as the fact that the spontaneous normalization of celiac specific antibodies is observed in some T1D patients, the search for new, early CD biomarkers in order to detect potential cases of CD is of key importance [22,23].

For this reason, we evaluated the utility of I-FABP—a recognized serological marker of intestinal epithelium damage in CD—as an early marker in T1D patients. To our knowledge, this is the first study describing such a relationship in a pediatric cohort.

The current study shows that the concentration of I-FABP is significantly elevated both in CD and T1D patients in comparison to healthy individuals, indicating small intestinal epithelium damage in both patient cohorts. However, there were no differences between the study groups, suggesting that the gut leakage, measured by I-FABP concentration, can be an independent predictor of CD development. This result was confirmed by the analysis of the ROC curve, based on which it can be concluded that I-FABP is not a good diagnostic marker of CD in T1D patients. This biomarker offers no ability to separate the two clinical conditions. The statistically determined optimal cut-off value of I-FABP for active CD at the level of 965 pg/mL produced sensitivity and specificity of only 51.7 and 59.7%, respectively. The analysis of the I-FABP concentrations in T1D-CD-1 in relation to T1D showed that even in the period preceding the onset of CD, its levels were elevated. This result clearly indicates that damage to the intestinal epithelial barrier is independent of the presence of CD.

These results support the previous hypothesis of the loss of intestinal barrier integrity in T1D resulting in low-grade chronic inflammation as well as increased diffusion of bacterial components into the blood. Previous studies showed intestinal barrier dysfunction assessed by measuring blood markers of intestinal damage or bacterial translocation other than I-FABP [24,25,26]. Elevated levels of zonulin [27,28,29], cytokeratin 18 caspase-cleaved fragment [30], lipopolysaccharides [28], and peptidoglycans [31] indicate both increased paracellular permeability and a profound damage to the intestine, allowing bacterial components to enter the bloodstream. Vaarala pointed out that the concentration of zonulin, the protein regulating the functioning of epithelial tight junctions, correlated with increasing intestinal permeability measured by the functional lactulose/mannitol test [26,27,32].

It is known that GFD is an effective treatment for celiac patients, inducing clinical recovery, the normalization of histopathological changes in the small intestine, and the normalization of serum autoantibody levels [33,34]. In this study, we found that GFD used for a minimum of 6 months decreased the concentration of I-FABP in CD and T1DM-CD patients by at least 50%. This finding indicates that gut barrier integrity can be significantly improved with proper dietary management. Interestingly, GFD in T1D-CD patients induced a I-FABP decrease, achieving the level observed in healthy controls. However, this observation does not answer the question of whether GFD may be effective in T1D patients without CD. The hypothesis that gluten is harmful not only to patients with CD but also to those with other autoimmune diseases was suggested recently [33,35]. Researchers suggest that GFD may offer the potential to reduce the risk of T1D [36], and a few studies indicate that GFD, when applied to older children with T1D, may protect beta cells from destruction to some extent. Undoubtedly, various factors determine the impact of GFD on the autoimmune response of pancreatic islets detected at the time of CD diagnosis: adherence to GFD and its duration, the type and concentration of anti-diabetic autoantibodies, and the asymptomatic clinical picture of CD [37].

While GFD can improve the intestinal epithelium in diabetic patients with CD, it is still an open question whether GFD could exert beneficial effects on the intestinal barrier in the early stages of T1D, protecting patients from the development of other autoimmune disorders, including CD, or impacting the clinical course of T1D.

## 5. Limitations and Strengths

We recognize some limitations of our study. First of all, it was limited by the relatively small number of patients. Secondly, we did not perform a sample size estimation. This is why when we found no difference between the study subgroups, it was difficult to determine whether this lack of difference was caused by the sample size. However, we believe that it is worth highlighting in our research that, due to access to such a broad clinical database (over a six-year period), it was possible to identify T1D patients who developed CD as a comorbid disease during this period. Thus, it was possible to retrospectively assess the concentration of I-FABP in the period of T1D only, one year before the appearance of CD serological markers (T1D-CD-1 group) and after classification in the T1D-CD group (after CD diagnosis). Moreover, the strength of this study is that all the T1D patients were serologically screened for celiac disease.

## 6. Conclusions

In summary, the evidence from this study suggests that I-FABP cannot serve as a potential early biomarker for diagnosis of CD in T1D patients, but it can be used as a serological marker indicating epithelial damage in pediatric T1D.

## Figures and Tables

**Table 1:** Characteristics of main study group: type 1 diabetes (T1D), type 1 diabetes with celiac disease (T1D-CD), celiac disease (CD). Separate subgroups: celiac disease on gluten-free diet (CD-GFD), type 1 diabetes with celiac disease on gluten-free diet (T1D-CD-GFD), and healthy controls (HC).

	Study Group (n = 245)			Control Group (n = 55)
Cohort	T1D	T1D-CD (T1D-CD-GFD)	CD (CD-GFD)	HC
Sample size	156	51 (39)	38 (36)	55
Gender				
Female	83	28 (20)	24 (22)	27
Male	73	23 (19)	14 (14)	28
Mean age in years	12	7 (7)	8 (8)	10
Mean age of T1D onset in years	9	6 (6)	NA (NA)	NA

T1D—type 1 diabetes, T1D-CD—type 1 diabetes and celiac disease, CD—celiac disease, CD-GFD—celiac disease on gluten-free diet, T1D-CD-GFD—type 1 diabetes and celiac disease on gluten-free diet, HC—healthy controls, NA—not applicable.

## Data Availability

The data presented in this study are available on request from the corresponding author. The data are not publicly available due to privacy protections.

## References

[1] Pham-Short A., Donaghue K.C., Ambler G., Phelan H., Twigg S., Craig M.E. (2015). Screening for Celiac Disease in Type 1 Diabetes: A Systematic Review. Pediatrics.

[2] Cerutti F., Bruno G., Chiarelli F., Lorini R., Meschi F., Sacchetti C. (2004). Diabetes Study Group of the Italian Society of Pediatric Endocrinology and Diabetology. Younger age at onset and sex predicts celiac disease in children and adolescents with type 1 diabetes: An Italian multicenter study. Diabetes Care.

[3] Wędrychowicz A., Minasyan M., Pietraszek A., Centkowski J., Stręk M., Różańska J., Chełmecka K., Zdzierak B., Wilk M., Czekańska P. (2021). Increased prevalence of celiac disease and its clinical picture among patients with diabetes mellitus type 1—Observations from a single pediatric center in Central Europe. Pediatr. Endocrinol. Diabetes Metab..

[4] Lettre G., Rioux J.D. (2008). Autoimmune diseases: Insights from genome-wide association studies. Hum. Mol. Genet..

[5] Achury J.G., Romanos J., Bakker S.F., Kumar V., de Haas E.C., Trynka G., Ricaño-Ponce I., Steck A., Chen W.M., Type 1 Diabetes Genetics Consortium (2015). Contrasting the genetic background of type 1 diabetes and celiac disease autoimmunity. Diabetes Care.

[6] Smyth D.J., Plagnol V., Walker N.M., Cooper J.D., Downes K., Yang J.H., Howson J.M., Stevens H., McManus R., Wijmenga C. (2008). Shared and distinct genetic variants in type 1 diabetes and celiac disease. N. Engl. J. Med..

[7] Zhernakova A., Withoff S., Wijmenga C. (2013). Clinical implications of shared genetics and pathogenesis in autoimmune diseases. Nat. Rev. Endocrinol..

[8] Cukrowska B., Sowińska A., Bierła J.B., Czarnowska E., Rybak A., Grzybowska-Chlebowczyk U. (2017). Intestinal epithelium, intraepithelial lymphocytes and the gut microbiota—Key players in the pathogenesis of celiac disease. World J. Gastroenterol..

[9] Akirov A., Pinhas-Hamiel O. (2015). Co-occurrence of type 1 diabetes mellitus and celiac disease. World J. Diabetes.

[10] Husby S., Koletzko S., Korponay-Szabó I.R., Mearin M.L., Phillips A., Shamir R., Troncone R., Giersiepen K., Branski D., Catassi C. (2012). European Society for Pediatric Gastroenterology, Hepatology, and Nutrition guidelines for the diagnosis of coeliac disease. J. Pediatr. Gastroenterol. Nutr..

[11] Mahmud F.H., Elbarbary N.S., Fröhlich-Reiterer E., Holl R.W., Kordonouri O., Knip M., Simmons K., Craig M.E. (2018). ISPAD Clinical Practice Consensus Guidelines 2018: Other complications and associated conditions in children and adolescents with type 1 diabetes. Pediatr. Diabetes.

[12] Oldenburger I.B., Wolters V.M., Kardol-Hoefnagel T., Houwen R.H.J., Otten H.G. (2018). Serum intestinal fatty acid-binding protein in the noninvasive diagnosis of celiac disease. APMIS.

[13] Ho S.S.C., Keenan J.I., Day A.S. (2020). The role of gastrointestinal-related fatty acid-binding proteins as biomarkers in gastrointestinal diseases. Dig. Dis. Sci..

[14] Adriaanse M.P.M., Mubarak A., Riedl R.G., Ten Kate F.J.W., Damoiseaux J.G.M.C., Buurman W.A., Houwen R.H.J., Vreugdenhil A.C.E., Celiac Disease Study Group (2017). Progress towards non-invasive diagnosis and follow-up of celiac disease in children; a prospective multicentre study to the usefulness of plasma I-FABP. Sci. Rep..

[15] Derikx J.P., Vreugdenhil A.C., Van den Neucker A.M., Grootjans J., van Bijnen A.A., Damoiseaux J.G., van Heurn L.W., Heineman E., Buurman W.A. (2009). A pilot study on the noninvasive evaluation of intestinal damage in celiac disease using I-FABP and L-FABP. J. Clin. Gastroenterol..

[16] Adriaanse M.P., Tack G.J., Passos V.L., Damoiseaux J.G., Schreurs M.W., van Wijck K., Riedl R.G., Masclee A.A., Buurman W.A., Mulder C.J. (2013). Serum I-FABP as marker for enterocyte damage in coeliac disease and its relation to villous atrophy and circulating autoantibodies. Aliment. Pharmacol. Ther..

[17] Hotamisligil G.S., Bernlohr D.A. (2015). Metabolic functions of FABPs—Mechanisms and therapeutic implications. Nat. Rev. Endocrinol..

[18] Lau E., Marques C., Pestana D., Santoalha M., Carvalho D., Freitas P., Calhau C. (2016). The role of I-FABP as a biomarker of intestinal barrier dysfunction driven by gut microbiota changes in obesity. Nutr. Metab..

[19] Pelsers M.M., Namiot Z., Kisielewski W., Namiot A., Januszkiewicz M., Hermens W.T., Glatz J.F. (2003). Intestinal-type and liver-type fatty acid-binding protein in the intestine. Tissue distribution and clinical utility. Clin. Biochem..

[20] Clinical Diabetology (2018). 2018 Guidelines on the management of diabetic patients. A position of Diabetes Poland. Clin. Diabet..

[21] Sun S., Puttha R., Ghezaiel S., Skae M., Cooper C., Amin R., Northwest England Paediatric Diabetes Network (2009). The effect of biopsy-positive silent coeliac disease and treatment with a gluten-free diet on growth and glycaemic control in children with Type 1 diabetes. Diabet. Med..

[22] Sud S., Marcon M., Assor E., Palmert M.R., Daneman D., Mahmud F.H. (2010). Celiac disease and pediatric type 1 diabetes: Diagnostic and treatment dilemmas. Int. J. Pediatr. Endocrinol..

[23] Unal E., Demiral M., Baysal B., Ağın M., Devecioğlu E.G., Demirbilek H., Özbek M.N. (2021). Frequency of celiac disease and spontaneous normalization rate of celiac serology in children and adolescent patients with type 1 diabetes. J. Clin. Res. Pediatr. Endocrinol..

[24] Mønsted M.Ø., Falck N.D., Pedersen K., Buschard K., Holm L.J., Haupt-Jorgensen M. (2021). Intestinal permeability in type 1 diabetes: An updated comprehensive overview. J. Autoimmun..

[25] Li X., Atkinson M.A. (2015). The role for gut permeability in the pathogenesis of type 1 diabetes—A solid or leaky concept?. Pediatr. Diabetes.

[26] Vaarala O. (2008). Leaking gut in type 1 diabetes. Curr. Opin. Gastroenterol..

[27] Sapone A., de Magistris L., Pietzak M., Clemente M.G., Tripathi A., Cucca F., Lampis R., Kryszak D., Cartenì M., Generoso M. (2006). Zonulin upregulation is associated with increased gut permeability in subjects with type 1 diabetes and their relatives. Diabetes.

[28] Leiva-Gea I., Sánchez-Alcoholado L., Martín-Tejedor B., Castellano-Castillo D., Moreno-Indias I., Urda-Cardona A., Tinahones F.J., Fernández-García J.C., Queipo-Ortuño M.I. (2018). Gut microbiota differs in composition and functionality between children with type 1 diabetes and mody2 and healthy control subjects: A case-control study. Diabetes Care.

[29] Wood Heickman L.K., DeBoer M.D., Fasano A. (2020). Zonulin as a potential putative biomarker of risk for shared type 1 diabetes and celiac disease autoimmunity. Diabetes Metab. Res. Rev..

[30] Hoffmanová I., Sánchez D., Hábová V., Anděl M., Tučková L., Tlaskalová-Hogenová H. (2015). Serological markers of enterocyte damage and apoptosis in patients with celiac disease, autoimmune diabetes mellitus and diabetes mellitus type 2. Physiol. Res..

[31] Duan Y., Prasad R., Feng D., Beli E., Li Calzi S., Longhini A.L.F., Lamendella R., Floyd J.L., Dupont M., Noothi S.K. (2019). Bone marrow-derived cells restore functional integrity of the gut epithelial and vascular barriers in a model of diabetes and ACE2 deficiency. Circ. Res..

[32] Bosi E., Molteni L., Radaelli M.G., Folini L., Fermo I., Bazzigaluppi E., Piemonti L., Pastore M.R., Paroni R. (2006). Increased intestinal permeability precedes clinical onset of type 1 diabetes. Diabetologia.

[33] Aljada B., Zohni A., El-Matary W. (2021). The gluten-free diet for celiac disease and beyond. Nutrients.

[34] Yoosuf S., Makharia G.K. (2019). Evolving therapy for celiac disease. Front. Pediatr..

[35] Lerner A., Freire de Carvalho J., Kotrova A., Shoenfeld Y. (2021). Gluten-free diet can ameliorate the symptoms of non-celiac autoimmune diseases. Nutr. Rev..

[36] Haupt-Jorgensen M., Holm L.J., Josefsen K., Buschard K. (2018). Possible prevention of diabetes with a gluten-free diet. Nutrients.

[37] Kaur P., Agarwala A., Makharia G., Bhatnagar S., Tandon N. (2020). Effect of gluten-free diet on metabolic control and anthropometric parameters in type 1 diabetes with subclinical celiac disease: A randomized controlled trial. Endocr. Pract..

